# Supplementary material for: Association of NUDT17 rs9286836 and rs2004659 variants with breast cancer risk in Bangladeshi Women
Source: PLoS One. 2026 Mar 19;21(3):e0344584. doi: 10.1371/journal.pone.0344584 (PMC13001948; doi:10.1371/journal.pone.0344584)
Supplement: S1 Table — This table summarizes socio-demographic and selected reproductive and lifestyle characteristics of breast cancer cases and healthy controls, including religion, gap between first and second child, duration of oral contraceptive pill use, history of postmenopausal hormonal therapy, smoking status, and family history of cancer. Group comparisons were performed using appropriate statistical tests, and corresponding p-values are shown. (DOCX) [file pone.0344584.s001.docx]

**S1 Table. Socio-demographic and clinical characteristics of breast cancer patients and controls.**

| **Types of Features** | **Case, n (%)** | **Control, n (%)** | **P Value** |
| --- | --- | --- | --- |
| **Religion** | | | |
| **Islam** | 218 (90.83) | 233 (97.08) | - |
| **Hindu** | 22 (9.17) | 6 (2.50) |  |
| **Christian** | 0 (0) | 1 (0.42) |  |
| **Gap Between 1^st^ and 2^nd^ Child (years)** | | | |
| **≤2** | 64 (33.16) | 47 (40.17) | 0.213 |
| **>2** | 129 (66.84) | 70 (59.83) |  |
| **Duration of Taking Contraceptive Pills (years)** | | | |
| **≤5** | 92 (61.74) | 88 (67.69) | 0.301 |
| **>5** | 57 (38.26) | 42 (32.31) |  |
| **History of Postmenopausal Hormonal Therapy** | | | |
| **Yes** | 2 (0.83) | 2 (0.83) | - |
| **No** | 238 (99.17) | 238 (99.17) |  |
|  | **Smoking** |  |  |
| **Yes** | 0 (0.0) | 2 (0.83) | - |
| **No** | 240 (100.0) | 238 (99.17) |  |
| **Family History of Cancer** | | | |
| **Yes** | 45 (18.75) | 33 (13.75) | 0.139 |
| **No** | 195 (81.25) | 207 (86.25) |  |
