# Supplementary figures and images for: Association of NUDT17 rs9286836 and rs2004659 variants with breast cancer risk in Bangladeshi Women
Source: PLoS One. 2026 Mar 19;21(3):e0344584. doi: 10.1371/journal.pone.0344584 (PMC13001948; doi:10.1371/journal.pone.0344584)

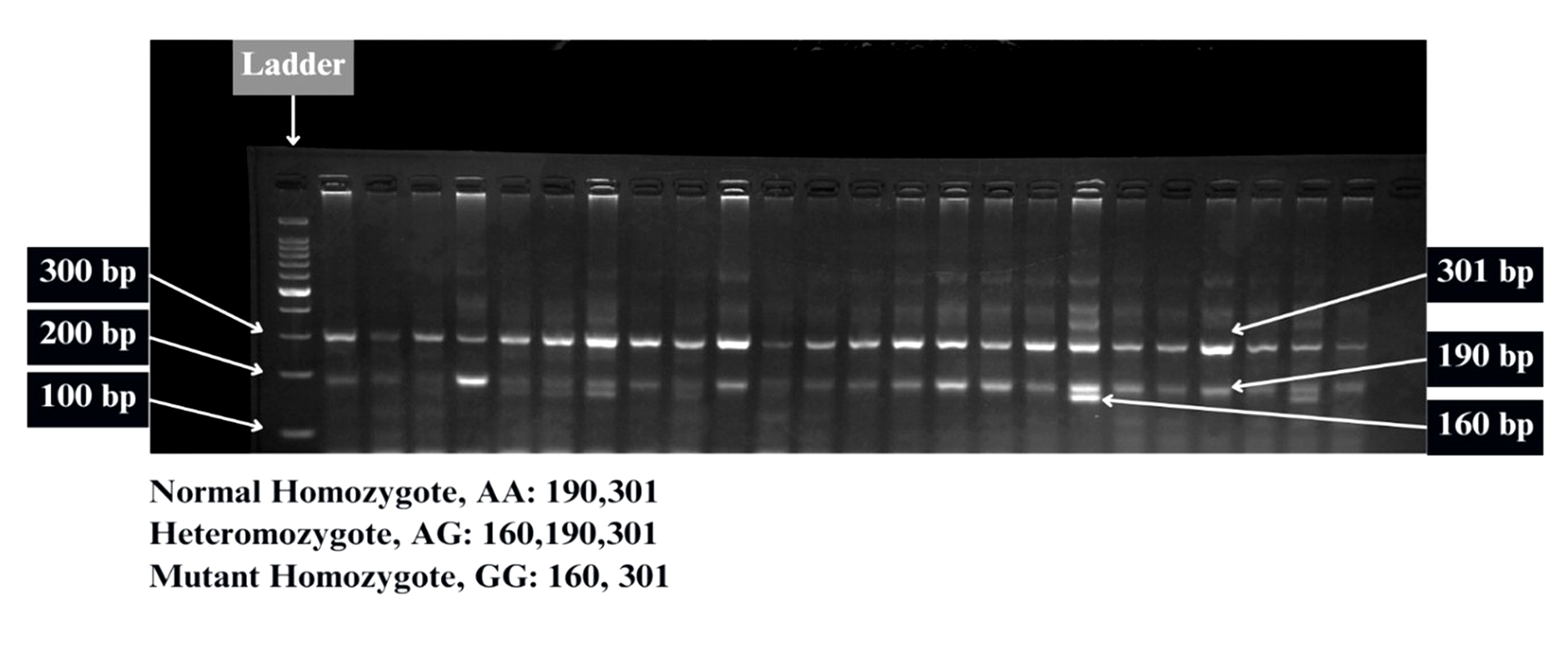

Supplement: S1 Fig — All lanes represent individual study samples, with Lane 1 containing a 100 bp DNA ladder. Selected lanes are annotated to illustrate representative genotypes: AA genotypes show bands at 190 bp and 301 bp, AG genotypes show bands at 160 bp, 190 bp, and 301 bp, and GG genotypes show bands at 160 bp and 301 bp. Band sizes correspond to the expected amplicon lengths. (TIF) [file pone.0344584.s002.tif]

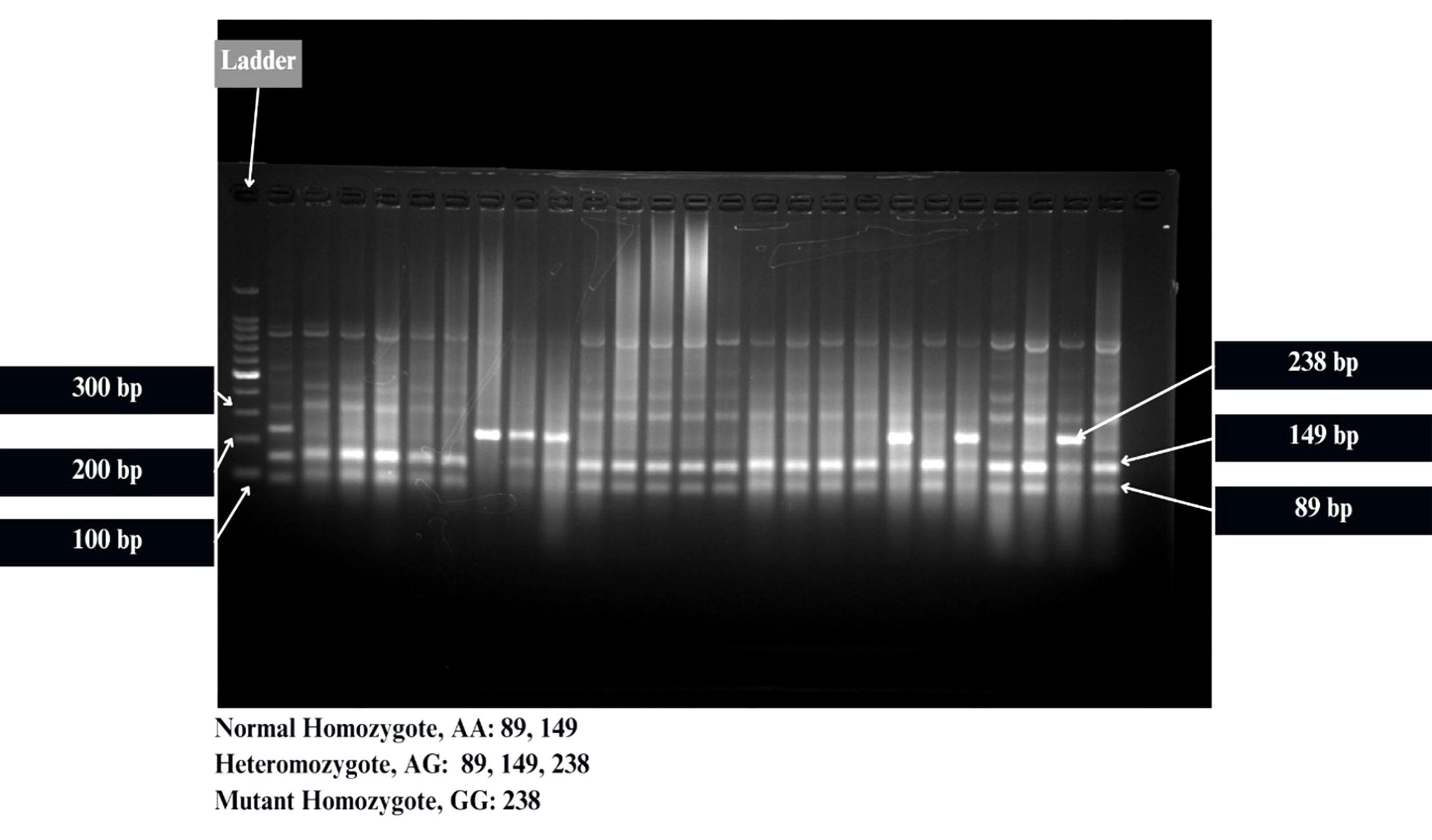

Supplement: S2 Fig — All lanes represent individual study samples, with Lane 1 containing a 100 bp DNA ladder. Selected lanes are annotated to illustrate representative genotypes: the AA genotype shows fragments at 89 bp and 149 bp, the AG genotype shows fragments at 89 bp, 149 bp, and 238 bp, and the GG genotype shows an undigested fragment at 238 bp. Fragment sizes correspond to the expected restriction patterns. (TIF) [file pone.0344584.s003.tif]
