# Supplementary material for: Association of NUDT17 rs9286836 and rs2004659 variants with breast cancer risk in Bangladeshi Women
Source: PLoS One. 2026 Mar 19;21(3):e0344584. doi: 10.1371/journal.pone.0344584 (PMC13001948; doi:10.1371/journal.pone.0344584)
Supplement: Attachment — Submitted filename: Review Report_PONE-D-25-57284.pdf [file pone.0344584.s005.pdf]

Reviewer Report
Manuscript Title:
Association of NUDT17 rs9286836 and rs2004659 Variants with Breast Cancer Risk in Bangladeshi Women
Article Type: Research Article

1. General Comments

The study is well-designed with comprehensive data collection and appropriate analysis. The study provides valuable preliminary data on breast cancer genetic susceptibility in Bangladeshi women. However, several sections need some clarification and improvements.

2. Specific Comments

Abstract

- This section is clearly described but needs to avoid excessive inclusion of numerical values from results and rather focus on significant findings.
- Try and narrative results for logistic regression then long OR/CIs numbers.

Introduction

- This section is detailed and backed with relevant literature. As for the section of *NUDIX* gene, the authors must focus on its role specifically with regards to breast cancer.
- Need to strengthen the justification for selecting rs9286836 and rs2004659 SNPs and emphasize the need for this analysis in Bangladeshi women.

Materials and Methods

Methods are well-detailed however, need to clarify;

- Selection criteria for study subjects and whether cases/controls were matched.
- Include genotype call validation (e.g., random re-genotyping for QC).
- In the sample size estimation paragraph do include actual sample sizes.
- In statistical analysis do indicate how *Hardy–Weinberg equilibrium* was tested. Also clarify *Bonferroni correction* (pre-specified or post hoc). If possible do consider adjusting logistic regression models adjusted for potential confounders (e.g., age, BMI, reproductive factors) for stronger outcomes.

Results

- Overall results are well organized but could be meaningful by summarizing them with relevant details.
- Data analysis presented in tables is comprehensive and well structured but needs to highlight statistically significant results e.g. of rs2004659 SNP and avoid overinterpretation for rs9286836 SNP.
- Gel electrophoresis figures are more suited to the supplementary section.

Discussion

- The results interpretation is well aligned and descriptive but avoid emphasis on some of the non-significant results and speculative statements.
- The *functional implications* of rs2004659 results need to be carefully discussed as the analyses are *in silico* and not experimentally validated.
- Inclusion of a study limitations paragraph will strengthen the manuscript.
- It's worth comparing the Bangladeshi population with the Chinese cohort but do focus on the potential genetic variability of both ethnic groups.
- In the conclusion section, avoid repeating results and focus on the main findings.

Language and Style

- The English is fluent and formal, but a few sentences are long and could be simplified.
- Maintain consistent use of "breast cancer" instead of abbreviations (BC) unless used frequently in tables.
- Check punctuation, spacing, and consistent decimal formatting (e.g., "p = 0.0098" rather than "p=0.0098").
- Ensure consistent use of scientific italics for gene symbols (*NUDT17*).

Figures and Tables

- Figures quality could be improved by enhancing resolution and clearer labelling.
- The rarely used sociodemographic variables presented in table 2 could be moved to Supplementary materials.

References

- All references are properly selected and up-to-date.
- Authors must ensure PLOS ONE style formatting.
- Citing papers on role of *NUDIX* enzymes to oxidative stress response in cancer will be a plus.

4. Recommendation

Decision: *Minor to moderate revision*
