# Supplementary material for: Association of NUDT17 rs9286836 and rs2004659 variants with breast cancer risk in Bangladeshi Women
Source: PLoS One. 2026 Mar 19;21(3):e0344584. doi: 10.1371/journal.pone.0344584 (PMC13001948; doi:10.1371/journal.pone.0344584)
Supplement: Attachment — Submitted filename: 30.11.2025-Review Report_Plos One.docx [file pone.0344584.s006.docx]

PONE-D-24-22063R1

Association of Vitamin D levels and Vitamin D receptor gene polymorphism with obesity in Bangladeshi school-going children: A cross-sectional study

PLOS ONE

My comments:

This is a very interesting study which will attract the attention of scientists and readers globally. However, manuscript requires a major revision and can be considered for publication after addressing the following comments.

1. In abstract authors require to add this sentence “An association between the NUDT17 polymorphisms rs9286836 and rs2004659 and the risk of breast cancer in Bangladeshi Women has not been studied previously and thus, this study aimed to determine this correlation. It will clarify the novelty of the study.
2. How was the number of BC participants determined? Sample size is small and should be at least 400 or more to be statistically valid according to the existing number of BC patients in Bangladesh. Please justify.
3. There are four SNPs; rs9286836, rs2004659, rs10910830, and rs10910829 probable binding determinants associated with the regulation of NUDT17 gene expression. Why only two SNPs rs9286836, rs2004659 were studied? I would recommend investigating the association of rs10910830, and rs10910829 with BC risk as well.
4. Genotyping was performed using tetra-primer ARMS-PCR for rs9286836 and PCR–RFLP for rs2004659, why two different techniques were used? Please explain.
5. Hardy-Weinberg equilibrium should be determined for allelic and genotypic model of each SNP. Include this in the statistical evaluation section and its findings in the result section.
6. In the introduction, please include the study conducted in Chinese women by Han et all., 2024 to reveal this association as a first line of the last paragraph.
7. In the discussion, include detail on allelic association with BC risk from the study conducted by Han et all, 2024 (Title of the study: **Association between *NUDT17* polymorphisms and breast cancer risk)** and compare your study findings with their findings.
8. In the discussion section, please include references for this statement “Previous studies on the NUDT17 rs9286836 and rs2004659 polymorphisms suggested a possible correlation between these genetic variants and cancer susceptibility”.
9. In the in the first line of conclusion, please replace “individuals” by “women”.

Reviewer: Dr Raushanara Akter

Professor, School of Pharmacy

BRAC University, Bangladesh
