# Supplementary material for: Association of NUDT17 rs9286836 and rs2004659 variants with breast cancer risk in Bangladeshi Women
Source: PLoS One. 2026 Mar 19;21(3):e0344584. doi: 10.1371/journal.pone.0344584 (PMC13001948; doi:10.1371/journal.pone.0344584)
Supplement: Attachment — Submitted filename: Response to reviewers.docx [file pone.0344584.s009.docx]

PONE-D-25-57284

Association of NUDT17 rs9286836 and rs2004659 Variants with Breast Cancer Risk in Bangladeshi Women

PLOS One

**Editor:**

Please include the following items when submitting your revised manuscript:

- A rebuttal letter that responds to each point raised by the academic editor and reviewer(s). You should upload this letter as a separate file labeled 'Response to Reviewers'.
- A marked-up copy of your manuscript that highlights changes made to the original version. You should upload this as a separate file labeled 'Revised Manuscript with Track Changes'.
- An unmarked version of your revised paper without tracked changes. You should upload this as a separate file labeled 'Manuscript'.

2. Thank you for stating the following financial disclosure:

“The study was partially financially supported by the Department of Pharmacy at the University of Asia Pacific.”

Please state what role the funders took in the study.  If the funders had no role, please state: "The funders had no role in study design, data collection and analysis, decision to publish, or preparation of the manuscript."

If this statement is not correct you must amend it as needed.

Please include this amended Role of Funder statement in your cover letter; we will change the online submission form on your behalf.

3. Please note that funding information should not appear in any section or other areas of your manuscript. We will only publish funding information present in the Funding Statement section of the online submission form. Please remove any funding-related text from the manuscript.

4. We note that there is identifying data in the Supporting Information file < Supplementary materials.rar>. Due to the inclusion of these potentially identifying data, we have removed this file from your file inventory. Prior to sharing human research participant data, authors should consult with an ethics committee to ensure data are shared in accordance with participant consent and all applicable local laws.

Data sharing should never compromise participant privacy. It is therefore not appropriate to publicly share personally identifiable data on human research participants. The following are examples of data that should not be shared:

-Name, initials, physical address

-Ages more specific than whole numbers

-Internet protocol (IP) address

-Specific dates (birth dates, death dates, examination dates, etc.)

-Contact information such as phone number or email address

-Location data

-ID numbers that seem specific (long numbers, include initials, titled “Hospital ID”) rather than random (small numbers in numerical order)

Data that are not directly identifying may also be inappropriate to share, as in combination they can become identifying. For example, data collected from a small group of participants, vulnerable populations, or private groups should not be shared if they involve indirect identifiers (such as sex, ethnicity, location, etc.) that may risk the identification of study participants.

Additional guidance on preparing raw data for publication can be found in our Data Policy (https://journals.plos.org/plosone/s/data-availability#loc-human-research-participant-data-and-other-sensitive-data) and in the following article: http://www.bmj.com/content/340/bmj.c181.long.

Please remove or anonymize all personal information (<specific identifying information in file to be removed>), ensure that the data shared are in accordance with participant consent, and re-upload a fully anonymized data set. Please note that spreadsheet columns with personal information must be removed and not hidden as all hidden columns will appear in the published file.

5. Please include captions for your Supporting Information files at the end of your manuscript, and update any in-text citations to match accordingly. Please see our Supporting Information guidelines for more information: http://journals.plos.org/plosone/s/supporting-information.

6. If the reviewer comments include a recommendation to cite specific previously published works, please review and evaluate these publications to determine whether they are relevant and should be cited. There is no requirement to cite these works unless the editor has indicated otherwise. 

**Response:**

The revised manuscript has been prepared in accordance with the Editor's and reviewers' suggestions. The patients’ and controls' IDs have been removed from the ‘Research data’ file and provided with just numbering as patient and control IDs. Funder information has been moved to the cover letter.

Reviewer's Responses to Questions

**Comments to the Author**

1. Is the manuscript technically sound, and do the data support the conclusions?

The manuscript must describe a technically sound piece of scientific research with data that supports the conclusions. Experiments must have been conducted rigorously, with appropriate controls, replication, and sample sizes. The conclusions must be drawn appropriately based on the data presented.

Reviewer #1: Yes

Reviewer #2: Yes

Reviewer #3: Yes

2. Has the statistical analysis been performed appropriately and rigorously?

Reviewer #1: No

Reviewer #2: No

Reviewer #3: Yes

3. Have the authors made all data underlying the findings in their manuscript fully available?

The [PLOS Data policy](http://www.plosone.org/static/policies.action#sharing) requires authors to make all data underlying the findings described in their manuscript fully available without restriction, with rare exception (please refer to the Data Availability Statement in the manuscript PDF file). The data should be provided as part of the manuscript or its supporting information, or deposited to a public repository. For example, in addition to summary statistics, the data points behind means, medians and variance measures should be available. If there are restrictions on publicly sharing data—e.g. participant privacy or use of data from a third party—those must be specified.

Reviewer #1: Yes

Reviewer #2: Yes

Reviewer #3: Yes

4. Is the manuscript presented in an intelligible fashion and written in standard English?

PLOS ONE does not copyedit accepted manuscripts, so the language in submitted articles must be clear, correct, and unambiguous. Any typographical or grammatical errors should be corrected at revision, so please note any specific errors here.

Reviewer #1: Yes

Reviewer #2: Yes

Reviewer #3: Yes

5. Review Comments to the Author

Please use the space provided to explain your answers to the questions above. You may also include additional comments for the author, including concerns about dual publication, research ethics, or publication ethics. (Please upload your review as an attachment if it exceeds 20,000 characters)

Reviewer #1: 1. General Comments
The study is well-designed with comprehensive data collection and appropriate analysis. The study provides valuable preliminary data on breast cancer genetic susceptibility in Bangladeshi women. However, several sections need some clarification and improvements.

**Response:**

We appreciate the positive evaluation. Following the reviewer’s suggestions, we have revised the Abstract, Introduction, Methods, Results, Discussion, and presentation of figures and tables to improve clarity, focus, and compliance with PLOS ONE standards.

2. Specific Comments
Abstract
• This section is clearly described but needs to avoid excessive inclusion of numerical values from results and rather focus on significant findings.
• Try and narrative results for logistic regression then long OR/CIs numbers.

**Response:**

Thank you for this comment. We have revised the abstract to reduce the emphasis on detailed numerical values and instead focus on the main findings that are most relevant biologically and clinically. The results are now described in a narrative manner, particularly for the logistic regression analyses, with emphasis placed on the statistically significant protective effect of the rs2004659 variant, rather than listing multiple odds ratios and confidence intervals.

**3. Introduction**
• This section is detailed and backed with relevant literature. As for the section of NUDIX gene, the authors must focus on its role specifically with regards to breast cancer.
• Need to strengthen the justification for selecting rs9286836 and rs2004659 SNPs and emphasize the need for this analysis in Bangladeshi women.

**Response:**

In addition, the introduction has been refined to better contextualize the role of the NUDT17 gene specifically in breast cancer. We have strengthened the rationale for selecting rs9286836 and rs2004659 by highlighting prior evidence, their potential functional relevance, and the lack of population-specific data. The need to evaluate these variants in Bangladeshi women is now more clearly stated, given the distinct genetic background and the limited existing data from this population.

4. Materials and Methods
Methods are well-detailed however, need to clarify;
• Selection criteria for study subjects and whether cases/controls were matched.
• Include genotype call validation (e.g., random re-genotyping for QC).
• In the sample size estimation paragraph do include actual sample sizes.
• In statistical analysis do indicate how Hardy–Weinberg equilibrium was tested. Also clarify Bonferroni correction (pre-specified or post hoc). If possible do consider adjusting logistic regression models adjusted for potential confounders (e.g., age, BMI, reproductive factors) for stronger outcomes.

**Response:**

We thank the reviewer for these valuable suggestions. We clarified the inclusion/exclusion criteria and explicitly stated age, sex, and BMI matching between cases and controls. Specifically, we have clarified the selection criteria and matching strategy for cases and controls, added a description of genotype call validation for quality control, explicitly stated the actual sample size used in the a priori power calculation, and expanded the statistical analysis section to describe Hardy-Weinberg equilibrium testing and the pre-specified use of Bonferroni correction. We also clarified the rationale for reporting crude logistic regression models and addressed potential confounding.

5. Results
• Overall results are well organized but could be meaningful by summarizing them with relevant details.
• Data analysis presented in tables is comprehensive and well structured but needs to highlight statistically significant results e.g. of rs2004659 SNP and avoid overinterpretation for rs9286836 SNP.
• Gel electrophoresis figures are more suited to the supplementary section.

**Response:**

We appreciate the reviewer's constructive suggestions. The results section has been revised to provide a clearer summary of the major findings, with emphasis on the statistically significant association observed for rs2004659 and careful wording for the non-significant result for rs9286836. Interpretations for rs9286836 have been limited to descriptive statements without speculative inference. The gel electrophoresis figures (Figures 1 and 2) have been moved to the Supplementary Information (S1 Fig and S2 Fig) as recommended.

6. Discussion
• The results interpretation is well aligned and descriptive but avoid emphasis on some of the non-significant results and speculative statements.
• The functional implications of rs2004659 results need to be carefully discussed as the analyses are in silico and not experimentally validated.
• Inclusion of a study limitations paragraph will strengthen the manuscript.
• It’s worth comparing the Bangladeshi population with the Chinese cohort but do focus on the potential genetic variability of both ethnic groups.

**Response:**
Thank you for these comments. We have gone through the discussion and made several small but important changes. We reduced the attention given to rs9286836, where the results were not significant, and removed wording that could be taken as speculative.

We also made it clearer that the functional interpretation of rs2004659 comes from in silico expression data and has not yet been tested experimentally. A separate paragraph on study limitations was added to explain the constraints of the sample size and the use of public databases.

The comparison with Chinese studies was revised as well, so that it now reflects possible differences between the two populations rather than assuming the same genetic effects. These changes were made to keep the discussion accurate and appropriately cautious.

7. In the conclusion section, avoid repeating results and focus on the main findings.

**Response:**

The conclusion section has been modified according to the reviewer’s suggestion.

8. Language and Style
• The English is fluent and formal, but a few sentences are long and could be simplified.
•Maintain consistent use of “breast cancer” instead of abbreviations (BC) unless used frequently in tables.
• Check punctuation, spacing, and consistent decimal formatting (e.g., “p = 0.0098” rather than “p=0.0098”).
• Ensure consistent use of scientific italics for gene symbols (NUDT17).

**Response**

We carefully reviewed the entire manuscript to improve readability and consistency. Several long or complex sentences were simplified without altering their scientific meaning. The term **“breast cancer”** is now used consistently throughout the text, with abbreviations avoided except where necessary in tables. Punctuation, spacing, and decimal formatting were standardized across the manuscript (e.g., p = 0.0098), and gene symbols, including NUDT17, were checked to ensure consistent use of scientific italics.

9. Figures and Tables
• Figures quality could be improved by enhancing resolution and clearer labelling.
• The rarely used sociodemographic variables presented in table 2 could be moved to Supplementary materials.

**Response:**

All figures were revised to improve resolution and visual clarity, with clearer labels and legends to enhance interpretability. Additionally, these figures have now been moved to the supplementary materials, as per your previous suggestion. In response to the suggestion regarding Table 2, sociodemographic variables that were not directly relevant to the genetic association analyses were moved to the **Supplementary Materials (S1Table),** allowing the main tables to focus on variables central to the study objectives.

10. References
• All references are properly selected and up-to-date.
• Authors must ensure PLOS ONE style formatting.
• Citing papers on role of NUDIX enzymes to oxidative stress response in cancer will be a plus.

**Response:**

In response to the reviewer’s suggestion, the manuscript already cites key studies describing the role of NUDIX family enzymes in oxidative stress and nucleotide sanitization, including foundational biochemical work on MTH1/MTH2 and oxidized nucleotide metabolism [24,30,31], as well as recent evidence linking NUDT17 polymorphisms to breast cancer susceptibility [25].

**Reviewer #2: PONE-D-24-22063R1**
Association of Vitamin D levels and Vitamin D receptor gene polymorphism with obesity in Bangladeshi school-going children: A cross-sectional study
PLOS ONE

My comments:
This is a very interesting study which will attract the attention of scientists and readers globally. However, manuscript requires a major revision and can be considered for publication after addressing the following comments.

**Response:**

Thank you very much for your comments. Although the title is not the title of our manuscript, all the comments are relevant to our manuscript.

We sincerely thank the reviewer for the constructive and detailed comments. Several of the points raised by you (Reviewer #2) overlap with those raised by Reviewer #1 and have already been addressed in the revised manuscript with tracked changes. For clarity, we respond to each comment individually below and indicate where revisions were made.

1. In abstract authors require to add this sentence “An association between the NUDT17 polymorphisms rs9286836 and rs2004659 and the risk of breast cancer in Bangladeshi Women has not been studied previously and thus, this study aimed to determine this correlation. It will clarify the novelty of the study.

**Response:**

We agree with the reviewer. The novelty of the study has been explicitly clarified in the abstract by stating that the association between NUDT17 rs9286836 and rs2004659 polymorphisms and breast cancer risk has not previously been investigated in Bangladeshi women. This revision also addresses a similar point raised by Reviewer #1 and has been incorporated into the revised manuscript with tracked changes.

2. How was the number of BC participants determined? Sample size is small and should be at least 400 or more to be statistically valid according to the existing number of BC patients in Bangladesh. Please justify.

**Response:**
An a priori sample size estimation was performed in G*Power (v3.1.9.7) using a chi-square test of independence under a dominant model (AG+GG vs AA; df = 1, α = 0.05, power = 0.80). The effect size (Cohen’s w = 0.14) was estimated from pilot genotype frequencies. The required total sample size was N = 400 (200 cases and 200 controls). The final analyzed sample for rs2004659 consisted of 408 participants (204 breast cancer cases and 204 controls), meeting the predefined power requirement, whereas analyses of rs9286836 included the full recruited cohort (240 cases and 240 controls).

3. There are four SNPs; rs9286836, rs2004659, rs10910830, and rs10910829 probable binding determinants associated with the regulation of NUDT17 gene expression. Why only two SNPs rs9286836, rs2004659 were studied? I would recommend investigating the association of rs10910830, and rs10910829 with BC risk as well.

**Response**

We appreciate this suggestion. The rationale for selecting rs9286836 and rs2004659 has now been clearly stated in the introduction, emphasizing prior evidence of functional relevance, reported associations in other populations, and feasibility within the current study design. While additional SNPs (rs10910830 and rs10910829) are acknowledged in the literature, investigating them was beyond the scope of the present study. **These variants will be considered in future studies with expanded cohorts to explore further the role of** NUDT17 **polymorphisms in breast cancer risk.** This clarification also addresses a similar point raised by Reviewer #1 and has been incorporated into the revised manuscript.

4. Genotyping was performed using tetra-primer ARMS-PCR for rs9286836 and PCR–RFLP for rs2004659, why two different techniques were used? Please explain.

**Response:** We appreciate the reviewer’s query. The use of two different genotyping techniques was based on SNP-specific sequence characteristics and methodological feasibility. For rs9286836, no suitable restriction enzyme recognition site is available using commercially accessible enzymes; therefore, PCR–RFLP is not applicable for this variant. As a result, tetra-primer ARMS-PCR was selected as a reliable and widely used alternative for allele discrimination. In contrast, rs2004659 contains an appropriate restriction enzyme cutting site, allowing accurate genotyping using the PCR–RFLP method. This explanation has now been explicitly added to the Genotyping subsection of the Materials and Methods. This clarification also addresses a similar methodological point raised by Reviewer #1 and has been incorporated into the revised manuscript with tracked changes.

5. Hardy-Weinberg equilibrium should be determined for allelic and genotypic model of each SNP. Include this in the statistical evaluation section and its findings in the result section.

**Response:** We agree with the reviewer that an assessment of Hardy-Weinberg equilibrium (HWE) is an important quality control step. Accordingly, HWE was evaluated for **the genotype distributions of each SNP in the control group using a chi-square goodness-of-fit test;** the results are reported in the Results section.
As HWE is defined for genotype frequencies rather than allele frequencies, it was not assessed separately for the allelic model. This clarification has now been explicitly stated in the Statistical Evaluation section.

6. In the introduction, please include the study conducted in Chinese women by Han et all., 2024 to reveal this association as a first line of the last paragraph.

**Response:** Agreed. The study by Han et al. (2024) has been incorporated into the final paragraph of the introduction to provide background on prior findings among Chinese women and to contextualize the rationale for the present study.

7. In the discussion, include detail on allelic association with BC risk from the study conducted by Han et all, 2024 (Title of the study: Association between NUDT17 polymorphisms and breast cancer risk) and compare your study findings with their findings.

**Response:** We have revised the discussion to include a direct comparison with the Chinese cohort reported by Han et al. (2024). Specifically, we discuss the reported protective allelic effects of rs9286836 and rs2004659 in Chinese women and contrast these findings with our results, which replicate the protective association for rs2004659 but not for rs9286836. The discussion now explicitly emphasizes potential population-specific genetic variability that may account for these differences.

8. In the discussion section, please include references for this statement “Previous studies on the NUDT17 rs9286836 and rs2004659 polymorphisms suggested a possible correlation between these genetic variants and cancer susceptibility”.

**Response:** The relevant statements in the discussion are now supported adequately with citations, including Han et al. (2024) and additional NUDIX-related literature.

9. In the in the first line of conclusion, please replace “individuals” by “women”.

**Response:** Thank you very much for your comment, and we have revised the manuscript according to your suggestion. The wording in the conclusion has been corrected to consistently refer to women, in line with the study population.

**Reviewer #3:** In introduction section:
1. Please add the background information regarding the association of SNPs of interest with breast cancer seen in other ethnicity (if available).

**Response:**

Thank you very much for your comments, and we agree with the reviewer. Background information on prior associations of the NUDT17 rs9286836 and rs2004659 polymorphisms with breast cancer in other ethnic populations has now been incorporated into the introduction. Specifically, findings from a recent case–control study in Chinese women have been cited to summarize previously reported associations and to highlight ethnic variability in genetic susceptibility. This addition strengthens the rationale for examining these SNPs in the Bangladeshi population and has been included in the final paragraph of the Introduction with tracked changes.

2. Avoid using the information regarding the current study method in the last part of the introduction, shift them to methodology. State the objective only.

**Response:**

We agree with the reviewer. The final paragraph of the introduction has been revised to **remove methodological details.**

**Methodology:**
1. Why the own study data was not used during in silico gene expression analysis?

**Response:**
We appreciate this important methodological question. In silico gene expression analyses were performed using publicly available, large-scale datasets (OncoDB and GTEx) because **gene expression profiling (e.g., RNA sequencing or microarray analysis)** was not conducted as part of the present study. Our study design focused on **genetic association analysis using germline DNA,** and no tumor or matched normal tissue samples were collected for transcriptomic assessment. Publicly curated datasets provide robust, well-annotated expression data derived from large cohorts and are commonly used to explore genotype-expression relationships when experimental expression data are not available. Accordingly, in silico analyses were used to provide supporting **functional context** rather than direct experimental validation. This clarification has now been explicitly stated in the Methodology section to avoid ambiguity.

**Result:**
2. It is better to describe as 'no association' for rs9286836 in stead of mentioning 'associated but not statistically significant'.

**Response:**

Thank you very much for your valuable comment. And we agree with the reviewer. The Results section has been revised to describe rs9286836 as showing no association with breast cancer risk, rather than implying an association that did not reach statistical significance. All descriptions of rs9286836 have been carefully reworded to present the findings clearly and conservatively, without speculative interpretation. These changes have been incorporated into the revised manuscript with tracked changes.

Overall:
1. Please write the full form when first using the acronyms/abbreviations.
2. I wonder why you have no co-authors from NICRH. How did you manage sample and data collection without collaborating with them?

**Response:** We agree with the reviewer. All acronyms and abbreviations are now written in full at their first occurrence in the manuscript, followed by the abbreviated form in parentheses. This revision has been applied consistently throughout the Abstract, main text, tables, and figure legends, and is visible in the revised manuscript with tracked changes.

We appreciate the reviewer’s concern regarding sample and data collection. Patient recruitment and sample collection were conducted at the National Institute of Cancer Research and Hospital (NICRH) with formal institutional permission and ethical approval. Clinical data and blood samples were collected by NICRH healthcare professionals as part of routine clinical procedures, and our researchers also assisted them. The study design, laboratory analyses, data analysis, and manuscript preparation were conducted by the authors at the University of Asia Pacific.
Accordingly, NICRH clinicians and staff contributed through institutional support and clinical facilitation rather than direct involvement in study design, analysis, or authorship-qualifying intellectual contributions, consistent with authorship guidelines. This clarification has now been explicitly stated in the **Materials and Methods (Participant Group / Ethical Clearance)** section to avoid ambiguity.
