# Supplementary material for: Association of NUDT17 rs9286836 and rs2004659 variants with breast cancer risk in Bangladeshi Women
Source: PLoS One. 2026 Mar 19;21(3):e0344584. doi: 10.1371/journal.pone.0344584 (PMC13001948; doi:10.1371/journal.pone.0344584)
Supplement: Attachment — Submitted filename: COMMENTS on Article (PONE-D-25-57284R1) _PlosOne_Dr. Akter.docx [file pone.0344584.s008.docx]

Manuscript Number: PONE-D-25-57284R1

Title of the manuscript: Association of NUDT17 rs9286836 and rs2004659 Variants with Breast Cancer Risk in Bangladeshi Women

Reviewer: Dr. Raushanara Akter, Professor, School of Pharmacy, BRAC University, Bangladesh

COMMENTS TO THE AUTHOR:

I would recommend accepting the manuscript for publication, as the authors have addressed all my comments and revised their manuscript accordingly.
